# Plasma metabolites associated with functional and clinical outcomes in heart failure with reduced ejection fraction with and without type 2 diabetes

**DOI:** 10.1038/s41598-022-12973-0

**Published:** 2022-06-02

**Authors:** Joseph B. Lerman, Stephanie N. Giamberardino, Adrian F. Hernandez, G. Michael Felker, Svati H. Shah, Robert W. McGarrah

**Affiliations:** 1 Division of Cardiology, Department of Medicine, Duke University School of Medicine, Durham, NC USA; 2 Duke Molecular Physiology Institute, Duke University School of Medicine, 300 N. Duke St, Durham, NC 27701 USA; 3 Duke Clinical Research Institute, Durham, NC USA

**Keywords:** Metabolomics, Biomarkers, Cardiology

## Abstract

Heart failure with reduced ejection fraction (HFrEF) is increasingly treated with medications for type 2 diabetes mellitus (T2DM). Whether metabolic derangements in HFrEF and T2DM are associated with differential outcomes remains unclear. Therefore, understanding molecular pathways in HFrEF and T2DM and their effects on clinical endpoints is important. The FIGHT trial randomized 300 individuals with HFrEF and a recent HF hospitalization to liraglutide (a GLP-1 receptor agonist) versus placebo to assess effects on mortality, HF rehospitalization, and 6-month change in NT-ProBNP. Although the trial showed no clinical benefit of liraglutide, the trial population was highly enriched for individuals with T2DM. Sixty metabolites were quantified via mass spectrometry in plasma from 254 FIGHT participants (N = 147 (57.9%) with T2DM). Principal components analysis reduced the high number of correlated metabolites into uncorrelated factors. The association of factor levels with 90-day changes in 6-min walk distance (6MWD) and NT-proBNP, and with time to mortality or HF hospitalization were evaluated. There were no changes in metabolite factors according to treatment assignment. However, in analyses stratified by T2DM status, changes in five plasma metabolite factors correlated with changes in functional outcomes beyond adjustment: factor 2 (branched-chain amino acids [BCAA]) correlated with changes in NT-proBNP (ρ = − 0.291, p = 4 × 10^–4^) and 6MWD (ρ= 0.265, p = 0.011); factor 1 (medium-chain acylcarnitines;  ρ = 0.220, p = 0.008), factor 4 (long-chain dicarboxylacylcarnitines; ρ = 0.191, p = 0.019), factor 5 (long-chain acylcarnitines; ρ = 0.198, p = 0.017), and factor 8 (urea cycle metabolites; ρ = − 0.239, p = 4 × 10^–3^), correlated with change in NT-proBNP. Factor 4 was associated with time-to-event (HR = 1.513 [95% CI 1.208–1.896], p = 3 × 10^–4^) with a trend towards stronger prognostic effect in T2DM (T2DM: p = 1 × 10^–3^, non-T2DM: p = 0.1). We identified metabolites of BCAA, urea cycle and fatty acid metabolism as biomarkers of HFrEF outcomes, with observed differences in HFrEF patients with T2DM. Such biomarkers might enable future diagnostic or therapeutic interventions in individuals with HFrEF and T2DM.

**Trial Registration:** Clinicaltrials.gov. Identifier: NCT01800968. First posted: February 28, 2013.

## Introduction

Type 2 diabetes mellitus (T2DM) is associated with incident HF^1^, and individuals with heart failure with reduced ejection fraction (HFrEF) and T2DM have increased morbidity and mortality compared to individuals with either diagnosis alone^2^. Like T2DM, HFrEF is characterized by alterations in energy homeostasis and impaired cardiac and systemic metabolism^3^. Recent work has demonstrated that T2DM medications, including GLP1 receptor agonists and SGLT2 inhibitors, lead to improved cardiovascular outcomes^4,5^. Notably, for patients with HFrEF, such clinical benefit is evident in individuals with and without T2DM treated with SGLT2 inhibitors^6^. While these findings will continue to change practice patterns, the biomolecular mechanisms responsible for these clinical improvements are still being elucidated^7–9^. As such, understanding molecular pathways in HFrEF and T2DM, and their effects on both functional outcomes and hard clinical endpoints is critical.

Metabolomic profiling, the study of small molecule byproducts of cellular metabolism, is a powerful tool to provide insight into cardiovascular disease pathogenesis, diagnosis and prognosis^10–12^. However, to date, there have been few studies that describe the in vivo metabolic derangements that characterize HFrEF in patients with comorbid T2DM, as compared to those without T2DM. Even less is known about the association of particular dysregulated metabolic processes and clinical outcomes in these distinct populations. The FIGHT (Functional Impact of GLP-1 for Heart Failure Treatment) trial randomized individuals with HFrEF to liraglutide versus placebo. Although the trial showed no clinical benefit of liraglutide, the trial population was highly enriched for individuals with T2DM. We therefore sought to characterize circulating metabolites associated with both functional and clinical outcomes in patients with high-risk HFrEF with and without comorbid T2DM from the FIGHT clinical trial.

## Methods

### Study population

The FIGHT trial randomized 300 individuals with HFrEF to liraglutide versus placebo, and was conducted from 2013 to 2015. Details of this study population and primary analyses have been previously reported^13^. All patients were greater than 18 years old, with an established diagnosis of HFrEF with LVEF of 40% or lower during the preceding three months. Additionally, all patients had a recent (within 14 days of enrollment) hospitalization for an acute heart failure exacerbation despite pre-existing use of guideline directed medical therapies for heart failure therapy, and a pre-admission oral diuretic dose of at least 40 mg of furosemide (or equivalent). The primary endpoint was a global rank score in which patients were ranked across three hierarchical tiers: time to death, time to rehospitalization for heart failure, and time-averaged proportional change in N-terminal pro-B-type natriuretic peptide (NT-proBNP) level from baseline to 180 days. Secondary outcomes of interest included exercise capacity, as measured by distance walked during a 6-min walk (6MWD) test at baseline and at 90 days, as well as serum NT-proBNP at baseline and 90 days. T2DM was defined by a subject having an established prior diagnosis of T2DM prior to trial enrollment. Assessment of both glycated hemoglobin (Hgb A1c) and homeostatic model assessment of insulin resistance (HOMA-IR) was performed at time of enrollment, 30, 90, and 180 days post-randomization. 256 of 300 participants in the FIGHT trial consented for participation in a biomarker substudy. Banked, frozen, fasting plasma samples from these individuals were used for metabolomic profiling in both baseline and 90-day post-enrollment samples. The current study was approved by the Duke University Institutional Review Board. All experiments were performed in accordance with the Declaration of Helsinki. Informed consent was obtained from all participants.

### Laboratory methods

Targeted metabolomics was performed using a quantitative tandem flow injection mass spectrometry-based approach with inclusion of spike-in standards to determine levels of 45 acylcarnitines and 15 amino acids in stored, frozen, previously unthawed fasting plasma samples obtained at baseline and at 90 days during the study period, using methods that we have previously used^14,15^. Proteins were first removed by precipitation with methanol; aliquoted supernatants were dried and esterified with hot acidic methanol (acylcarnitines) and n-butanol (amino acids). For the analysis, we used tandem mass spectrometry with a Quattro Micro instrument (Waters Corp., Milford, Massachusetts), and the addition of internal standards enabled quantitative assessment of metabolites^15^. Testing for all of the assays was done in random batch order by the Metabolomics/Biomarker Core Laboratory of the Duke Molecular Physiology Institute at Duke University; testing personnel were blinded to the clinical status of patients, and samples were randomly distributed without knowledge of event status.

### Statistical analysis

**Figure 1:**
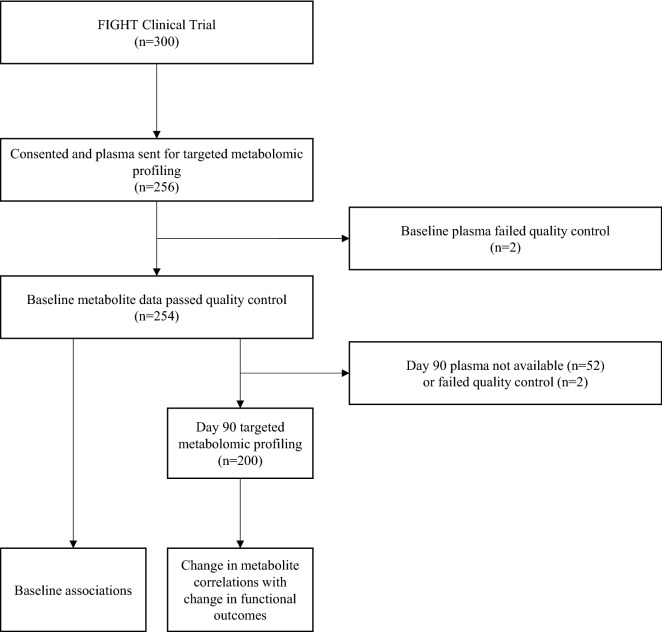
Flow diagram of patient selection.

Baseline characteristics were compared between individuals with T2DM and without T2DM using chi-square tests for categorical variables and Kruskal–Wallis rank sum tests for continuous variables. Two participants were excluded from analyses due to sample issues, and another participant’s day 90 sample was also excluded due to consistently having the lowest measurement for 13/15 profiled amino acids. This resulted in a dataset consisting of n = 254 baseline samples and n = 200 day 90 samples available for inclusion in analyses (Fig. 1). Our overall analytic approach was to use principal components analysis (PCA) for dimensionality reduction; discovery analyses were then conducted for the correlation between change in PCA factor with change in functional parameters (NT-proBNP and 6MWD), adjusted for multiple comparisons using the False Discovery Rate (FDR) of Benjamini-Hochberg. Significant PCA factors were then further analyzed for (1) association between baseline PCA factor level with change in functional outcomes (NT-proBNP and 6MWD); (2) event prediction using time-to-event, using a nominal p < 0.05; and (3) analyses stratified by T2DM status. Exploratory analyses and results are also presented for PCA metabolite factors that were nominally significant (unadjusted for multiple comparisons p < 0.05) in the discovery correlation analyses.

Specifically, PCA with varimax rotation was performed utilizing baseline-only metabolites and then baseline-only weights were used to calculate 90 day factors to reduce the large number of correlations between metabolites into 13 uncorrelated factors (Supplementary Table S1), as we have done previously^15^. Four metabolites (C6, C7-DC, C16:2, C18:2-OH) were excluded from PCA analysis due to > 25% zero values in the baseline-only dataset and were not further analyzed. Analyses were performed on factors meeting Kaiser criterion (Eigenvalue > 1)^16^. To eliminate the influence of extreme factor outliers, baseline and day 90 factor values ≥ 6 standard deviations away from the mean were set to missing and not included in downstream analyses. Analyses proceeded as follows: (1) Spearman correlation coefficients were calculated in discovery analyses across all PCA-derived metabolite factors, adjusted for multiple comparisons using FDR, for the correlation between 90-day change in functional outcomes with change in factors (done separately for change in NT-proBNP and for change in 6MWD); PCA factors with an FDR p value of < 0.05 in these discovery analyses were moved forward for subsequent analyses, including: (2) linear regression analyses for association between baseline PCA factors and change in functional outcomes (NT-proBNP, 6MWD) in basic (adjusted for baseline functional outcome, age, sex, race, treatment assignment (liraglutide vs. placebo), and T2DM) and full models (adjusted for baseline NT-proBNP, age, sex, race, treatment assignment, BMI, baseline creatinine, baseline systolic blood pressure, baseline liver function tests, and T2DM [and baseline 6MWD only in analyses involving 6MWD]); (3) time-to-event (death or heart failure hospitalization) analyses using Cox proportional hazards models in basic (adjusted for age, sex, race, treatment assignment & diabetes status) and full models (adjusted for age, sex, race, treatment assignment, BMI, baseline creatinine, baseline systolic blood pressure, baseline NT-proBNP, and baseline liver function tests); and (4) the above analyses focused on differences by T2DM status: (a) univariate linear regression to determine association of T2DM status with baseline metabolite factor, (b) Spearman correlation coefficients for change in factor with change in functional outcomes stratified by T2DM; (c) linear regression analyses for association between baseline factor levels with change in functional outcomes, stratified by T2DM; and (d) time-to-event analyses stratified by T2DM. In these analyses focused on T2DM, if visual differences between T2DM and non-T2DM were observed, an interaction term (metabolite factor*T2DM) was fit in the basic model to assess for statistical significance of the interaction. Fisher r-to-z transformation tests were used to determine differences in correlations in T2DM stratified analyses. Exploratory results are also presented for metabolite factors nominally significant in discovery analyses across all analyses. Linear and Cox proportional hazards model estimates are reported in un-standardized units. For Cox proportional hazards models, the proportional hazards assumption test p value for the factor model term is reported.

## Results

### Baseline participant characteristics

Baseline study participant characteristics are presented in Table 1. Individuals included in this biomarker substudy had similar age, sex, and racial/ethnic characteristics as the entire FIGHT cohort population (Supplementary Table S2). Participants with T2DM (n = 147) were older, had higher body mass index, longer duration of heart failure, and greater prevalence of ischemic heart disease and hypertension than participants without T2DM (n = 107). Individuals with and without T2DM had similar severity of HFrEF by NYHA heart failure classes, similar left ventricular ejection fraction, and similar usage of goal directed medical therapies (Table 1).

**Table 1:** Baseline characteristics of the study cohort.

Variable	Overall (n = 254)	T2DM (n = 147)	No T2DM (n = 107)	P-value
**Clinical variables**				
Age (years) (median [IQR])	61 [52, 68]	63 [55, 68]	57 [46, 65]	**0.001**
Female sex (%)	53 (21)	35 (24)	18 (17)	NS
White race (%)	152 (60)	89 (61)	63 (59)	NS
Hispanic (%)	13 (5)	9 (6)	4 (4)	NS
Body mass index (median [IQR])	32 [25, 37]	33 [28, 39]	30 [25, 35]	**0.006**
**NYHA classification (%)**				NS
2	77 (30)	43 (29)	34 (32)	
3	157 (62)	92 (63)	65 (61)	
4	12 (5)	8 (5)	4 (4)	
6-min walk distance (m) (median [IQR])	227 [145, 315]	214 [140, 306]	245 [149, 343]	NS
**Physical examination**				
Weight (kg) (median [IQR])	96 [78, 115]	97 [83, 118]	91 [75, 107]	**0.02**
Systolic blood pressure (mmHg) ((median [IQR])	108 [99, 118]	108 [99, 120]	106 [98, 116]	NS
Heart rate (bpm) (median [IQR])	75 [68, 86]	74 [68, 84]	77 [68, 88]	NS
Duration since diagnosis of heart failure (years) (median [IQR])	6 [3, 11]	8 [4, 13]	5 [3, 10]	**0.02**
**Medical history**				
Prior hospitalization for heart failure within past year (%)	221 (87)	130 (88)	91 (85)	NS
Ischemic heart disease (%)	212 (83)	130 (88)	82 (77)	**0.02**
Hypertension (%)	201 (79)	124 (84)	77 (72)	**0.03**
Atrial fibrillation (%)	122 (48)	75 (51)	47 (44)	NS
Type 2 diabetes mellitus (%)	147 (58)	147 (100)	0 (0)	** < 0.001**
Stage > = 3 chronic kidney disease (%)	98 (39)	55 (37)	43 (40)	NS
**Cardiovascular medications at enrollment**				
Beta-blocker (%)	237 (93)	138 (94)	99 (93)	NS
ACE-inhibitor or ARB (%)	181 (71)	108 (73)	73 (68)	NS
Hydralazine (%)	89 (35)	47 (32)	42 (39)	NS
Long-acting nitrates (%)	99 (39)	57 (39)	42 (39)	NS
Aldosterone antagonist (%)	154 (61)	88 (60)	66 (62)	NS
Loop diuretic (%)	252 (99)	146 (99)	106 (99)	NS
Digoxin (%)	90 (35)	48 (33)	42 (39)	NS
Calcium-channel blocker (%)	15 (6)	11 (7)	4 (4)	NS
Lipid-lowering agent (%)	185 (73)	125 (85)	60 (56)	** < 0.001**
Antiplatelet agent (%)	183 (72)	115 (78)	68 (64)	**0.02**
Anticoagulant agent (%)	141 (56)	84 (57)	57 (53)	NS
**Laboratory and echocardiographic measures**				
Creatinine (mg/dL) (median [IQR])	1 [1, 2]	2 [1, 2]	1 [1, 2]	0.05
HbA1c (%) (median [IQR])	7 [6, 8]	8 [7, 8]	6 [6, 6]	** < 0.001**
Total cholesterol (mg/dL) (median [IQR])	132 [110, 165]	132 [110, 162]	132 [110, 174]	NS
NT-proBNP (pg/mL) (median [IQR])	1961 [1056, 4339]	1798 [1008, 3252]	2672 [1184, 5442]	**0.03**
LVEF (%) (median [IQR])	25 [19, 32]	25 [19, 33]	25 [19, 32]	NS

Significant values are in bold.

### Correlation between change in metabolite factors and change in functional outcomes

PCA identified 13 metabolite factors grouping in biologically consistent pathways (Supplementary Table S2) and similarly to our prior studies^14,15^. In discovery analyses, changes in five metabolite factors were significantly correlated with changes in functional outcomes (change in NT-proBNP and change in 6MWD from baseline to 90 days) after adjustment for multiple comparisons at the level of number of analyzed factors (Tables 2 and 3). Change in factor 2 (composed of branched chain amino acids and related metabolites [BCAA] including valine, leucine/isoleucine, glutamine/glutamic acid and also alanine and proline) was correlated both with change in NT-proBNP (ρ = − 0.291, adjusted p = 4 × 10^–4^) and change in 6MWD (ρ = 0.265, adjusted p = 0.011). Change in factor 1 (composed of medium chain acylcarnitines; ρ = 0.220, adjusted p = 0.008), factor 4 (composed of long chain dicarboxylacylcarnitines; ρ = 0.191, adjusted p = 0.019), factor 5 (composed of long chain acylcarnitines; ρ = 0.198, adjusted p = 0.017), and factor 8 (composed of urea cycle metabolites [citrulline and ornithine] and proline; ρ = − 0.239, adjusted p = 0.004) correlated with change in NT-proBNP but none of these factors correlated with change in 6MWD (Tables 2 and 3; Supplementary Figs. S1, S2). Accounting for the directions of the effect and of the load of individual metabolites on the factor (i.e. individual metabolites can have negative loads on a given factor), this signifies that change in individual BCAA levels are negatively correlated with change in NT-proBNP and positively correlated with change in 6MWD; and that change in levels of individual urea cycle metabolites is negatively correlated with change in NT-proBNP while changes in medium chain acylcarnitines, long chain acylcarnitines and long chain dicarboxylacylcarnitines are positively correlated with change in NT-proBNP. Partial correlations adjusted for treatment assignment showed similar effects (Tables 2, 3).

**Table 2:** Spearman correlation analyses between 90-day change in metabolite factor level and 90-day change in NT-proBNP.

Factor	Correlation	Overall				T2DM				Non-T2DM				T2DM vs Non-T2DM
		P-value	Adj. P-value	Partial correlation^a^	Partial correlation P-value	Correlation	P-value	Partial correlation^a^	Partial correlation P-value	Correlation	P-value	Partial correlation^a^	Partial correlation P-value	P-value
1	**0.220**	**0.002**	**0.008**	**0.220**	**0.002**	0.076	0.419	0.075	0.423	**0.390**	**3 × 10**^**–4**^	**0.394**	**3 × 10**^**–4**^	0.022
2	**− 0.291**	**3 × 10**^**–5**^	**4 × 10**^**–4**^	**− 0.294**	**3 × 10**^**–5**^	**− 0.248**	**0.007**	**− 0.250**	**0.007**	**− 0.392**	**2 × 10**^**–4**^	**− 0.395**	**2 × 10**^**–4**^	0.269
3	0.017	0.814	0.831	0.016	0.819									
4	**0.191**	**0.007**	**0.019**	**0.191**	**0.007**	0.128	0.175	0.128	0.177	**0.264**	**0.016**	**0.271**	**0.014**	0.332
5	**0.198**	**0.005**	**0.017**	**0.196**	**0.006**	**0.215**	**0.020**	**0.218**	**0.019**	0.167	0.131	0.172	0.123	0.733
6	0.106	0.133	0.217	0.106	0.136									
7	0.102	0.151	0.218	0.101	0.156									
8	**− 0.239**	**0.001**	**0.004**	**− 0.240**	**0.001**	− 0.146	0.117	− 0.146	0.117	**− 0.328**	**0.002**	**− 0.327**	**0.003**	0.183
9	0.127	0.074	0.138	0.129	0.070									
10	− 0.097	0.173	0.225	− 0.096	0.177									
11	− 0.057	0.427	0.504	− 0.054	0.447									
12	0.015	0.831	0.831	0.014	0.847									
13	**0.147**	**0.039**	0.084	0.148	0.038	**0.217**	**0.019**	**0.218**	**0.019**	0.058	0.604	0.052	0.646	0.266

Significant values are in bold.

^a^Partial correlations adjusted for treatment assignment.

**Table 3:** Spearman correlation analyses between 90-day change in metabolite factor level and 90-day change in 6-minute walk distance.

Factor	Correlation	Overall				T2DM				Non-T2DM				T2DM vs Non-T2DM
		P-value	Adj. P value	Partial correlation^a^	Partial correlation P-value	Correlation	P-value	Partial correlation^a^	Partial correlation P-value	Correlation	P-value	Partial correlation^a^	Partial correlation P-value	P-value
1	− 0.012	0.886	0.912	− 0.011	0.890									
2	**0.265**	**0.001**	**0.011**	**0.264**	**0.001**	**0.308**	**0.003**	**0.308**	**0.003**	0.203	0.108	0.202	0.113	0.499
3	0.009	0.912	0.912	0.009	0.916									
4	0.073	0.369	0.768	0.074	0.368									
5	− 0.152	0.058	0.188	− 0.153	0.057									
6	− 0.070	0.385	0.768	− 0.071	0.383									
7	**− 0.178**	**0.026**	0.141	**− 0.179**	**0.026**	− 0.080	0.450	− 0.079	0.454	**− 0.292**	**0.019**	**− 0.283**	**0.025**	0.184
8	− 0.022	0.789	0.912	− 0.022	0.787									
9	0.058	0.473	0.768	0.06	0.459									
10	− 0.023	0.779	0.912	− 0.023	0.775									
11	**0.171**	**0.033**	0.141	**0.173**	**0.031**	**0.253**	**0.015**	**0.256**	**0.015**	0.107	0.400	0.104	0.419	0.363
12	0.060	0.457	0.768	0.06	0.459									
13	− 0.014	0.864	0.912	− 0.013	0.875									

Significant values are in bold.

^a^Partial correlations adjusted for treatment assignment.

In analyses stratified by T2DM, visually significant differences were also observed for factors 1, 2, 4, 5 and 8, with change in factor levels showing a stronger correlation with change in NT-proBNP in non-T2DM versus T2DM for factors 1, 2, 4 and 8; and a stronger correlation in T2DM versus non-T2DM for factor 5. However, the correlations were not significantly different (Table 2). Factor 2 showed a visually stronger correlation with 6MWD in T2DM versus non-T2DM, although the correlations were not significantly different (Table 3).

Other factors that were nominally significant (i.e. but not once FDR adjusted for multiple comparisons) included factor 7 (amino acids, change in 6MWD, p = 0.03); factor 11 (Asx—aspartate/asparagine, change in 6MWD, p = 0.03); and factor 13 (C5:1 acylcarnitine, change in NT-proBNP, p = 0.04).

### Baseline metabolite factors associated with change in functional outcomes

Of the five metabolite factors significant after FDR adjustment in these discovery analyses of correlation of change in metabolites with change in functional outcomes, baseline levels of factor 8 (urea cycle metabolites) were associated with change in NT-proBNP (basic model estimate 757.495, p = 0.005, full model estimate 607.423, p = 0.05), (Table 4), but were not associated with change in 6MWD (Table 5). In T2DM stratified analyses, factor 8 showed visually stronger effects in non-T2DM (full model estimate 1193.632), although the interaction was non-significant (p = 0.35). For other metabolite factors nominally associated in the above correlation analyses, baseline levels of factor 11 (Asx—aspartate/asparagine) and factor 13 (C5:1 acylcarnitine) were associated with change in 6MWD in basic and full models (Table 5).

**Table 4:** Association of baseline factor level with 90-Day change in NT-proBNP.

Factor	Overall estimate	P-value	Diabetic estimate	P-value	Non-diabetic estimate	P-value
**Basic**						
1	268.651	0.505	− 164.308	0.719	1228.465	0.104
2	− 228.426	0.387	− 291.548	0.348	− 25.689	0.958
4	− 174.252	0.737	− 204.213	0.763	− 311.809	0.703
5	65.615	0.791	− 222.873	0.459	495.399	0.242
7	− 15.614	0.951	− 588.263	0.057	749.912	0.086
8	**757.495**	**0.005**	514.046	0.100	**1222.194**	**0.013**
11	100.557	0.684	− 477.538	0.121	542.204	0.208
13	247.111	0.402	− 144.919	0.695	546.801	0.280
**Full**^**a**^						
8	607.423	0.052	435.181	0.252	**1193.632**	**0.042**

Significant values are in bold.

^a^Model: change in NT-proBNP (D90 – baseline) ~ Baseline factor + baseline NT-proBNP + age + sex + race + randomization drug + BMI + baseline creatinine + SBP + liver function tests + diabetes (overall only).

**Table 5:** Association of baseline factor level with 90-day change in 6-min walk distance (6MWD).

Factor	Overall estimate	P-value	Diabetic estimate	P-value	Non-diabetic estimate	P-value
**Basic**						
1	4.254	0.750	7.042	0.702	1.477	0.942
2	− 15.290	0.099	− 16.155	0.209	− 13.567	0.351
4	− 3.531	0.831	− 6.660	0.760	− 0.283	0.992
5	15.407	0.103	7.786	0.551	**31.156**	**0.032**
7	− 1.248	0.892	6.821	0.594	− 14.656	0.321
8	− 15.685	0.119	− 7.370	0.597	**− 32.856**	**0.033**
11	**− 26.923**	**0.004**	**− 30.528**	**0.017**	− 25.749	0.072
13	**− 25.391**	**0.032**	− 22.506	0.153	− 28.126	0.154
**Full**^**a**^						
11	**− 25.278**	**0.010**	− 24.562	0.088	− 31.830	0.060
13	**− 28.932**	**0.032**	− 28.366	0.114	− 33.403	0.173

Significant values are in bold.

^a^Model: change in 6 min walk dist (D90 − baseline) ~ baseline factor + baseline 6 min walk dist + age + sex + race + randomization drug + BMI + baseline creatinine + SBP + baseline NT-proBNP + liver function tests + diabetes (overall only).

### Baseline metabolite factors associated with incident events

**Figure 2:**
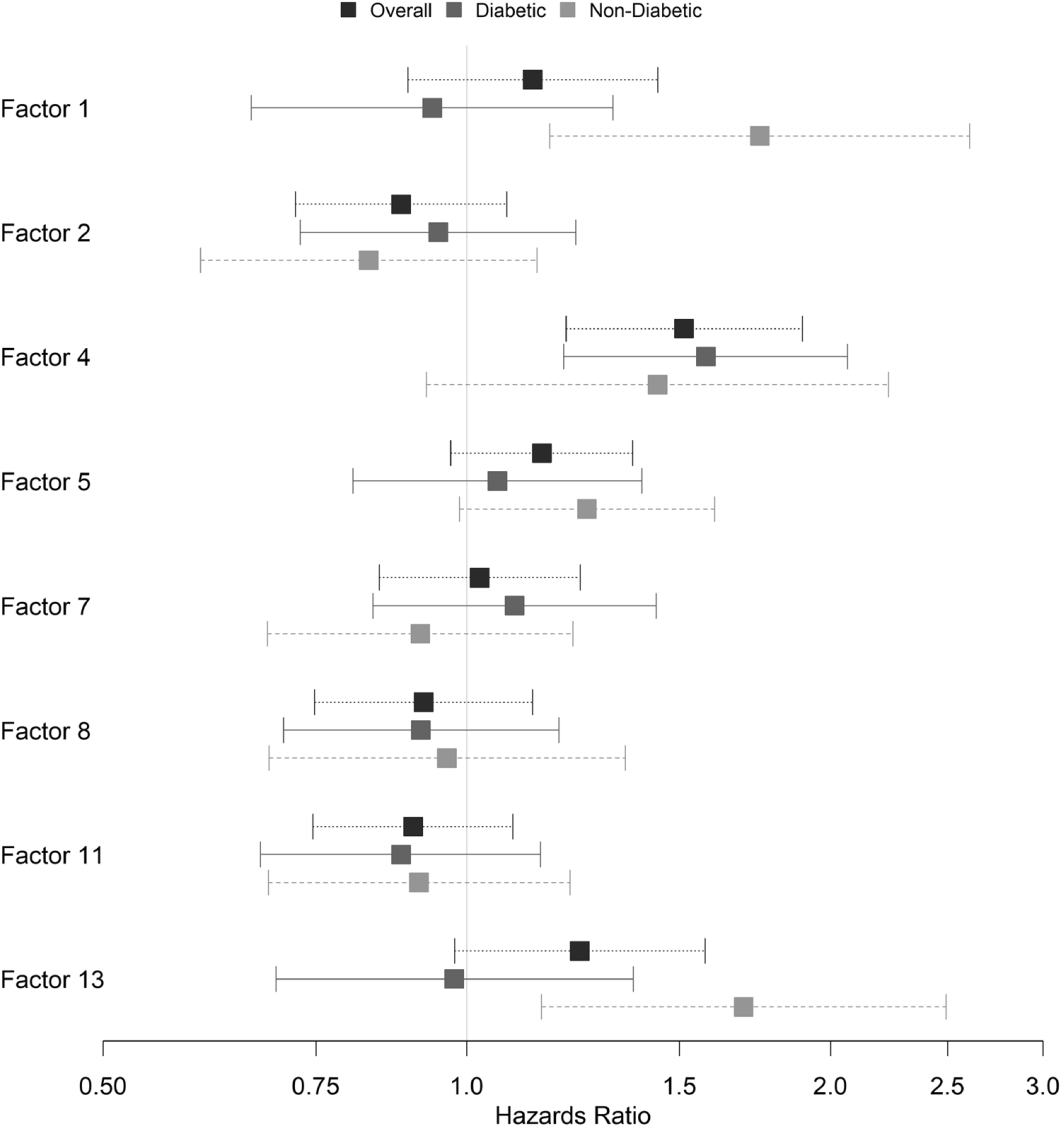
Forest plot of hazards ratios for baseline metabolite factor associations with time-to-event (death or heart failure hospitalization). Shown are hazards ratios for the overall cohort and those individuals with and without T2DM.

**Figure 3:**
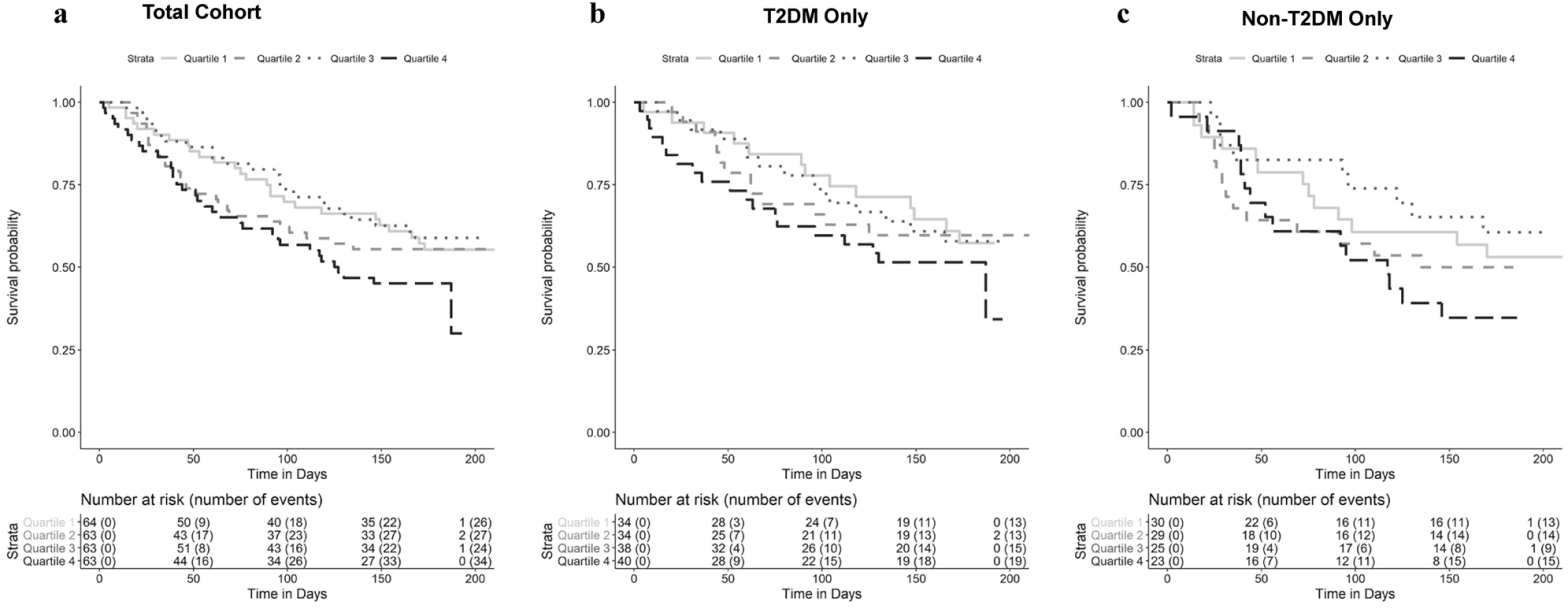
Time to death or heart failure hospitalization by quartile of factor 4 level. Kaplan–Meier curves stratified by quartiles of factor 4 levels in the overall cohort (**a**) and individuals with (**b**) and without (**c**) T2DM. The numbers below each graph represent the number of individuals at risk and the cumulative incidence of events at each time point in parentheses.

In Cox proportional hazards analyses for time to death or heart failure hospitalization, of the five factors associated with change in functional outcomes (Tables 2 and 3), only factor 4 (long chain dicarboxylacylcarnitines, LCDA) was significantly associated with time-to-event in basic (HR 1.51, p = 3 × 10^–4^) and full models (HR 1.50, p = 0.007) (Table 6, Fig. 2). Figure 3 shows univariate Kaplan–Meier curves for factor 4 in the overall cohort and in those with and without T2DM, with levels stratified into quartiles for visualization. Given the positive load of individual LCDA on factor 4, this signifies that the highest levels of LCDA at baseline were associated with an increased hazard of death or heart failure hospitalization, though this relationship was non-linear. In analyses stratified by T2DM, factor 4 was more significantly associated with time-to-event in individuals with T2DM (T2DM: basic model HR 1.58, p = 0.001; full model HR 1.58, p = 0.009; no-T2DM: basic model HR 1.44, p = 0.11; full model HR 1.07, p = 0.83) (Table 6, Fig. 2), although the interaction term added to the basic model was not significant (interaction p = 0.6).

**Table 6:** Time-to-event analyses for baseline factor levels and death or heart failure hospitalization.

Factor	Overall			Diabetic			Non-diabetic		
	HR (95% CI)	P-value	Prop. Haz. P-val	HR (95% CI)	P-value	Prop. Haz. P-val	HR (95% CI)	P-value	Prop. Haz. P-val
**Basic**									
1	1.13 (0.89–1.44)	0.300	0.436	0.94 (0.66–1.32)	0.707	0.691	**1.75 (1.17–2.61)**	**0.006**	0.659
2	0.88 (0.72–1.08)	0.222	0.859	0.95 (0.73–1.23)	0.683	0.321	0.83 (0.60–1.14)	0.252	0.751
4	**1.51 (1.21–1.90)**	**3 × 10**^**–4**^	0.506	**1.58 (1.20–2.07)**	**0.001**	0.414	1.44 (0.93–2.24)	0.106	0.697
5	1.15 (0.97–1.37)	0.106	0.056	1.06 (0.81–1.40)	0.678	0.031	1.26 (0.99–1.60)	0.065	0.381
7	1.03 (0.85–1.24)	0.801	0.673	1.10 (0.84–1.44)	0.510	0.360	0.92 (0.68–1.22)	0.551	0.847
8	0.92 (0.75–1.13)	0.437	0.550	0.92 (0.71–1.19)	0.514	0.538	0.96 (0.69–1.35)	0.829	0.923
11	0.90 (0.75–1.09)	0.292	0.642	0.88 (0.68–1.15)	0.355	0.334	0.91 (0.69–1.22)	0.534	0.604
13	1.24 (0.98–1.58)	0.077	0.841	0.98 (0.70–1.37)	0.891	0.386	**1.70 (1.15–2.49)**	**0.007**	0.338
**Full**^**a**^									
4	**1.50 (1.12–2.00)**	**0.007**	0.524	**1.58 (1.12–2.21)**	**0.009**	0.351	1.07 (0.61–1.87)	0.825	0.493

Significant values are in bold.

^a^Full model: time to heart failure hospitalization or death ~ baseline factor + age + sex + race + randomization drug + BMI + baseline creatinine + SBP + baseline NT-proBNP + liver function tests.

### Association between baseline metabolite factors with T2DM and treatment assignment

Although our full models adjusted for T2DM, to more explicitly visualize whether observed differences are accounted for by differences in metabolites between individuals with and without T2DM, we performed univariate linear regression of metabolite factors between these groups. None of the FDR-adjusted (factors 1, 2, 4, 5 and 8) nor nominally significant factors (factors 7 and 13) were associated with T2DM in these analyses except for factor 11 (composed of aspartate/asparagine) (Table 7).

**Table 7:** Association of metabolite factor levels with T2DM status.

Factor	Est	P-value
1	0.05	0.58
2	0.23	0.07
4	0.07	0.45
5	− 0.25	0.05
7	− 0.20	0.12
8	0.11	0.37
**11**	**− 0.28**	**0.03**
13	0.04	0.74

Significant values are in bold.

When we examined change in each metabolite factor stratified by treatment assignment, none of the factors had differential changes between the liraglutide and placebo arms.

## Discussion

Recent clinical trials have demonstrated that medications targeting T2DM, including GLP-1 receptor agonists and SGLT2 inhibitors, lead to improved cardiovascular outcomes in populations both with, and without concomitant T2DM^4,6^. While such findings have altered treatment strategies for HFrEF, the biomolecular mechanisms responsible for these clinical improvements are still being elucidated^7–9^. As such, understanding specific molecular pathways in HFrEF and T2DM, and their effects on both functional endpoints and clinical outcomes is critical. Leveraging a well-phenotyped clinical trial population of individuals with high-risk HFrEF enriched for T2DM, we have found peripheral blood metabolite signatures that associate with T2DM and with longitudinal changes in indicators of functional prognosis in HFrEF; and that independently predict clinical outcomes, a relationship that is modified by the presence of T2DM. Specifically, we have identified metabolic pathways that report on BCAA mitochondrial catabolism, urea cycle metabolism and fatty acid oxidation that are differentially associated with functional outcomes in HFrEF in individuals with and without T2DM; and metabolites that are likely byproducts of peroxisomal long chain fatty acid catabolism (long chain dicarboxylacylcarnitines, LCDA) that appear to be prognostic markers in individuals with T2DM.

BCAA (leucine, isoleucine, valine) are essential amino acids that play important roles in normal cellular growth as metabolic substrates and also via their impact on nutrient signaling pathways such as mTOR. An extensive body of literature from both human and animal studies has connected elevated levels of circulating BCAA and related metabolites from the BCAA catabolic pathway to the pathogenesis of cardiometabolic disorders—including obesity, insulin resistance, diabetes, and heart failure^17–21^. We found that change in a BCAA-related factor correlated with changes in both NT pro-BNP and 6MWD over a 90-day follow-up period, such that increases in this factor associated with decreases in NT pro-BNP and increases in 6MWD. Although these correlations remained significant in individuals with T2DM when the cohort was stratified by T2DM status, this factor was not associated with T2DM per se. These findings suggest that the correlation of the BCAA-related factor and improved functional outcomes is not necessarily related to underlying metabolic differences in individuals with and without T2DM. Rather, increases in BCAA may reflect other physiologic changes in individuals with T2DM and HFrEF that relate to improved functional outcomes. The FIGHT trial consisted of individuals with advanced HFrEF, with the majority of people exhibiting NYHA functional class 3–4 status and having been hospitalized for HF within the past year. As HF progresses, a catabolic/anabolic imbalance ensues, leading to systemic tissue wasting that is clinically most evident as progressive sarcopenia^22^. Circulating BCAA concentrations are associated with skeletal muscle mass and function in elderly populations, predict sarcopenia in individuals with cirrhosis, and positively correlate with skeletal muscle mass in individuals with HFrEF^23–26^. Thus, increases in plasma BCAA may represent T2DM-related improvement in skeletal muscle mass and performance in individuals with HFrEF that is reflected in decreased NT pro-BNP and increased 6MWD.

We also found that increases in a factor comprised of the urea cycle intermediates, citrulline and ornithine, the amino acid proline, and the short-chain dicarboxylacylcarntine [SCDA] C5-DC were associated with decreases in 90-day NT pro-BNP, an association that was stronger in individuals without T2DM. Similarly, baseline levels of this factor were associated with improvement in 90-day NT pro-BNP more strongly in individuals without T2DM. Proline and ornithine share biosynthetic pathways; and citrulline, which is formed from ornithine, is involved in nitric oxide synthesis. Impaired nitric oxide synthesis and signaling has been implicated in heart failure pathogenesis and progression and is also perturbed in T2DM^27–29^. Thus, our findings may report on dysregulated nitric oxide metabolism in comorbid HFrEF and T2DM that is reflected in the association of this metabolite factor with improved functional outcomes only in individuals without T2DM.

Circulating plasma acylcarnitines, which are intermediates of mitochondrial β-oxidation of fatty acids, are an emerging molecular signature of HF and are thought to reflect mitochondrial dysfunction^11,13,15^. We found that changes in factor 1 (medium chain acylcarnitines) and factor 5 (long chain acylcarnitines) correlated with changes in NT pro-BNP. Interestingly, factor 1 had a stronger correlation in individuals without T2DM while factor 5 had a stronger correlation in individuals with T2DM. Because medium and long chain fatty acids use different transport mechanisms into the mitochondria as well as different mitochondrial acyl-CoA dehydrogenase enzymes to generate downstream oxidative intermediates, the associations among individuals with and without T2DM may be related to these differences.

Factor 4, composed of long chain dicarboxylacylcarnitines (LCDA), was also associated with change in NT pro-BNP and was the only factor that was predictive of clinical outcomes (time to death or HF hospitalization), a finding that was stronger in individuals with T2DM. Fatty acid β-oxidation occurs in the mitochondria and is the primary means by which cells oxidize fatty acids in healthy states. However, in the setting of mitochondrial dysfunction that occurs in both HFrEF and T2DM, fatty acid oxidation may shift to other subcellular organelles, resulting in the reliance on peroxisomal β- and ω-oxidation, the latter of which generates LCDA as oxidative intermediates^30,31^. The comorbid conditions of HFrEF and T2DM, therefore, may contribute to circulating LCDA as markers of mitochondrial dysfunction that are prognostic for adverse events.

The overall result of the FIGHT trial was negative, which may explain the lack of association of treatment assignment with changes in metabolite factors that were associated with functional and clinical outcomes.

The strengths of our study include the use of samples collected as part of an adjudicated clinical trial with intermediate and hard clinical outcomes. We performed targeted metabolomic profiling which allows for precise quantification of metabolite concentrations that can facilitate use as clinical biomarkers. However, this targeted approach also limits our metabolome coverage, which is narrower than qualitative nontargeted metabolomics platforms. Additionally, our results should be interpreted in the context of this high-risk, high-morbidity patient population (67% NYHA class 3 or 4), and as such may not be generalizable to other HFrEF cohorts. Further studies should be performed to determine if these plasma metabolites correlate with longitudinal changes in cardiac structure and function. Finally, we report on peripheral blood metabolite profiles, and thus cannot assume that they represent myocardial processes alone, but rather are reflective of alterations in systemic metabolism.

## Conclusion

In conclusion, we have identified distinct peripheral metabolite profiles reporting on BCAA and urea cycle metabolism and mitochondrial dysfunction that differentially associate with functional and clinical outcomes in individuals with HFrEF and T2DM. These findings hold potential to further elucidate the pathophysiology of HFrEF in patients with T2DM and may allow for better risk stratification of this high-risk patient population.

## Supplementary Information

Supplementary Information.

## Data Availability

The clinical datasets analyzed during the current study are available by request in the BIOLINCC repository or by contacting the corresponding author of this study.
