# Supplementary material for: Plasma metabolites associated with functional and clinical outcomes in heart failure with reduced ejection fraction with and without type 2 diabetes
Source: Sci Rep. 2022 Jun 2;12:9183. doi: 10.1038/s41598-022-12973-0 (PMC9163122; doi:10.1038/s41598-022-12973-0)

**Supplementary Data**

# Plasma Metabolites Associated with Functional and Clinical Outcomes in Heart Failure with Reduced Ejection Fraction with and without Type 2 Diabetes


**Authors:** Joseph B. Lerman, MD[1], Stephanie N. Giamberardino, ScM[2], Adrian F. Hernandez, MD, MHS[1,3], G. Michael Felker, MD, MHS[1,3], Svati H. Shah, MD, MHS[1,2,3], Robert W. McGarrah, MD[1,2]

**Author Affiliations:**

1. Division of Cardiology, Department of Medicine, Duke University School of Medicine, Durham, NC
2. Duke Molecular Physiology Institute, Duke University School of Medicine, Durham, NC
3. Duke Clinical Research Institute, Durham, NC

**Supplementary Table S1: Principal Component Analysis Derived Metabolite Factors**

| Factor | Loaded Metabolites | Metabolite Loading Direction(s) |
|---|---|---|
| **1** (Medium & Long-chain Acylcarnitines) | C8, C5-DC, C10:1, C10, C12:1, C12, C12-OH/C10-DC, C14:2, C14:1, C14, C16:1, C16, C16:1-OH/C14:1-DC, C18 | (+) |
| **2** (Amino Acids) | Alanine, Proline, Valine, Leucine/Isoleucine, Glutamine/Glutamic Acid | (+) |
| **3** (Short-chain Dicarboxylacylcarnitines) | C4-DC/Ci4-DC, C8:1, C8:1-OH/C6:1-DC, C6-DC/C8-OH, C10:3, C10:2, C10:1, C8:1-DC | (+) |
| **4** (Long-chain Acylcarnitines) | C14-OH/C12-DC, C16-OH/C14-DC, C18:1-OH/C16:1-DC, C18-OH/C16-DC, C20, C18:1-DC, C20-OH/C18-DC | (+) |
| **5** (Arginine, Long-chain Acylcarnitines) | Arginine, C16, C18 :2, C18 :1, C18, C20 :4 | Arginine (-); All others (+) |
| **6** (Amino Acids) | Glycine, Serine, Methionine | (-) |
| **7** (Amino Acids) | Methionine, Histidine, Phenylalanine, Tyrosine, Ornithine | (+) |
| **8** (Amino Acids) | Proline, Ornithine, Citrulline, C5-DC | (+) |
| **9** (Misc Acylcarnitines) | C6-DC/C8-OH, C22 | C6-DC/C8-OH (+); C22 (-) |
| **10** (Acylcarnitines) | C2, C4-OH, C10-OH/C8-DC, C14:1-OH, C18:1-OH/C16:1-DC, C18:1-DC | (-) |
| **11** (Asparagine/Aspartic Acid) | Asparagine/Aspartic Acid | (+) |
| **12** (Short-chain Acylcarnitines) | C2, C3, C4/Ci4, C5, C5-OH/C3-DC, C4-DC/Ci4-DC | (-) |
| **13** (C5:1) | C5:1 | (-) |

**Supplementary Table S2: Baseline characteristics of metabolomics cohort as compared to total study population**

| Variable | Metabolomics Cohort (n=254) | Total Study Population (n=300) |
|---|---|---|
| **Clinical Variables** | | |
| Age (years) (median [IQR]) | 61 [52, 68] | 61 [52, 68] |
| Female sex (%) | 53 (21) | 64 (21) |
| White race (%) | 152 (60) | 172 (57) |
| Body mass index (median [IQR]) | 32 [25, 37] | 32 [26, 37] |
| NYHA Classification (%) | | |
| 2 | 77 (30) | 85 (28) |
| 3 | 157 (62) | 189 (63) |
| 4 | 12 (5) | 14 (5) |
| 6-min walk distance (m) (median [IQR]) | 227 [145, 315] | 223 [144, 311] |
| **Physical Examination** | | |
| Weight (kg) (median [IQR]) | 96 [78, 115] | 96 [79, 115] |
| Systolic blood pressure (mmHg) ((median [IQR]) | 108 [99, 118] | 108 [99, 119] |
| Heart rate (bpm) (median [IQR]) | 75 [68, 86] | 76 [68, 86] |
| Duration since diagnosis of heart failure (years) (median [IQR]) | 6 [3, 11] | 6 [3, 11] |
| **Medical History** | | |
| Prior hospitalization for heart failure within past year (%) | 221 (87) | 262 (87) |
| Ischemic heart disease (%) | 212 (83) | 246 (82) |

| | | |
|---|---|---|
| Hypertension (%) | 201 (79) | 235 (78) |
| Atrial fibrillation (%) | 122 (48) | 144 (48) |
| Type 2 diabetes mellitus (%) | 147 (58) | 178 (59) |
| Stage >= 3 chronic kidney disease (%) | 98 (39) | 118 (39) |
| **Laboratory and Echocardiographic Measures** | | |
| Creatinine (mg/dL) (median [IQR]) | 1 [1, 2] | 1 [1, 2] |
| HbA1c (%) (median [IQR]) | 7 [6, 8] | 7 [6, 8] |
| Total cholesterol (mg/dL) (median [IQR]) | 132 [110, 165] | 131 [109, 163] |
| NT-proBNP (pg/mL) (median [IQR]) | 1961 [1056, 4339] | 2049 [1054, 4235] |
| LVEF (%) (median [IQR]) | 25 [19, 32] | 25 [19, 32] |

**Supplementary Figure S1. Correlation plots of 90-day change in NT-proBNP and 90-day change in metabolite factor.** Plots are shown for the entire cohort and for individuals with and without T2DM for metabolite factors that had statistically significant associations with 90-day change in NT-proBNP (**a-f**).

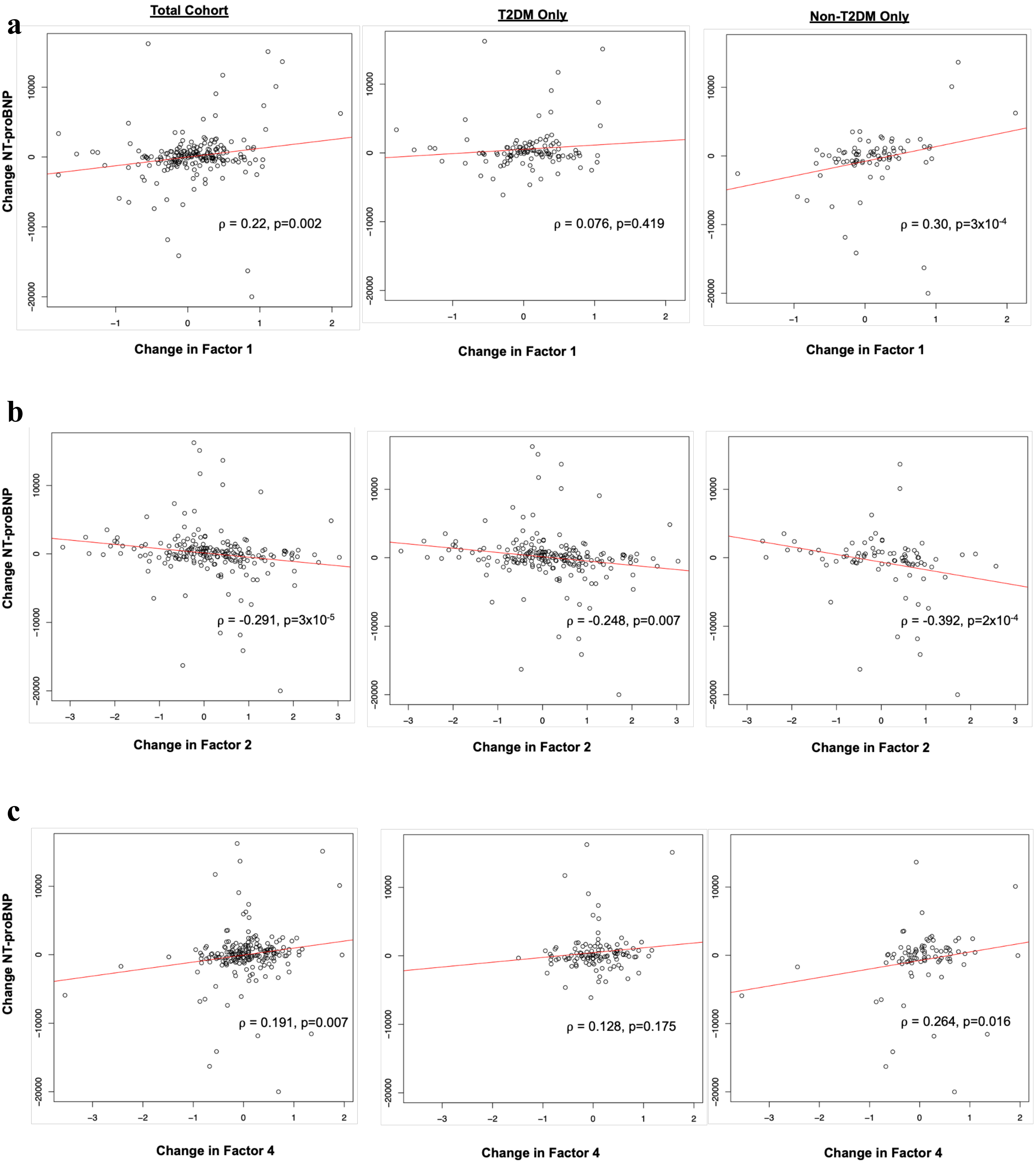

d

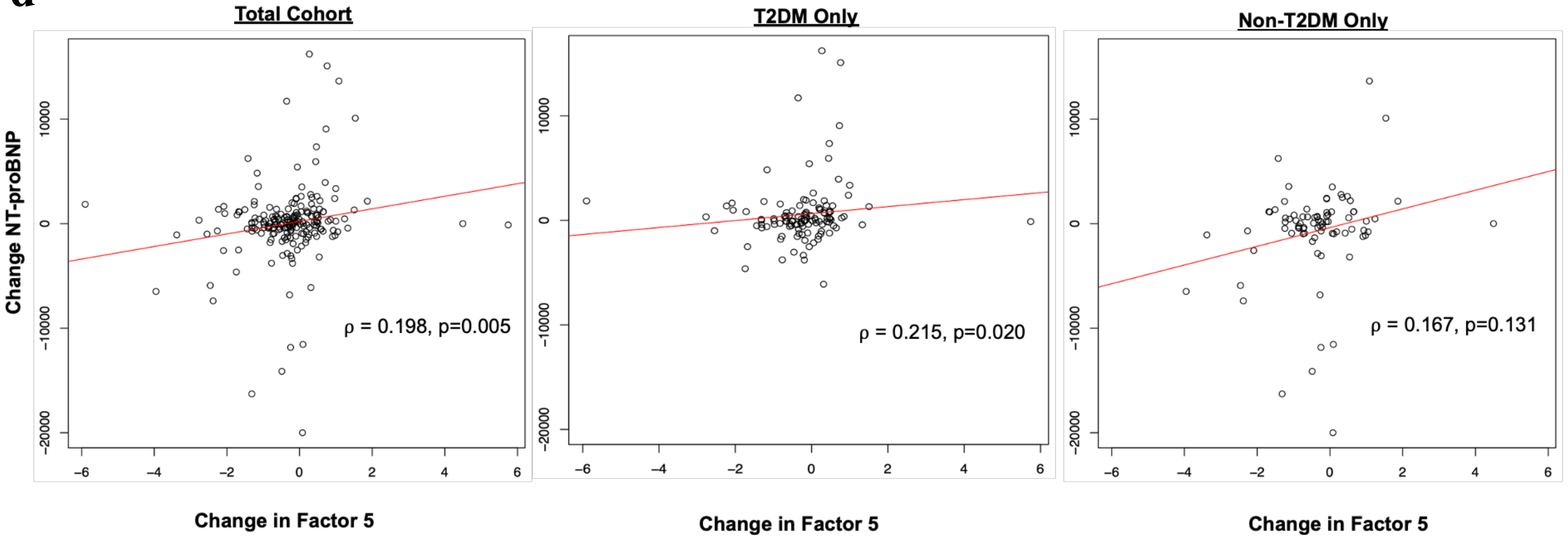
Total Cohort
T2DM Only
Non-T2DM Only
Change NT-proBNP
ρ = 0.198, p=0.005
ρ = 0.215, p=0.020
ρ = 0.167, p=0.131
Change in Factor 5
Change in Factor 5
Change in Factor 5

e

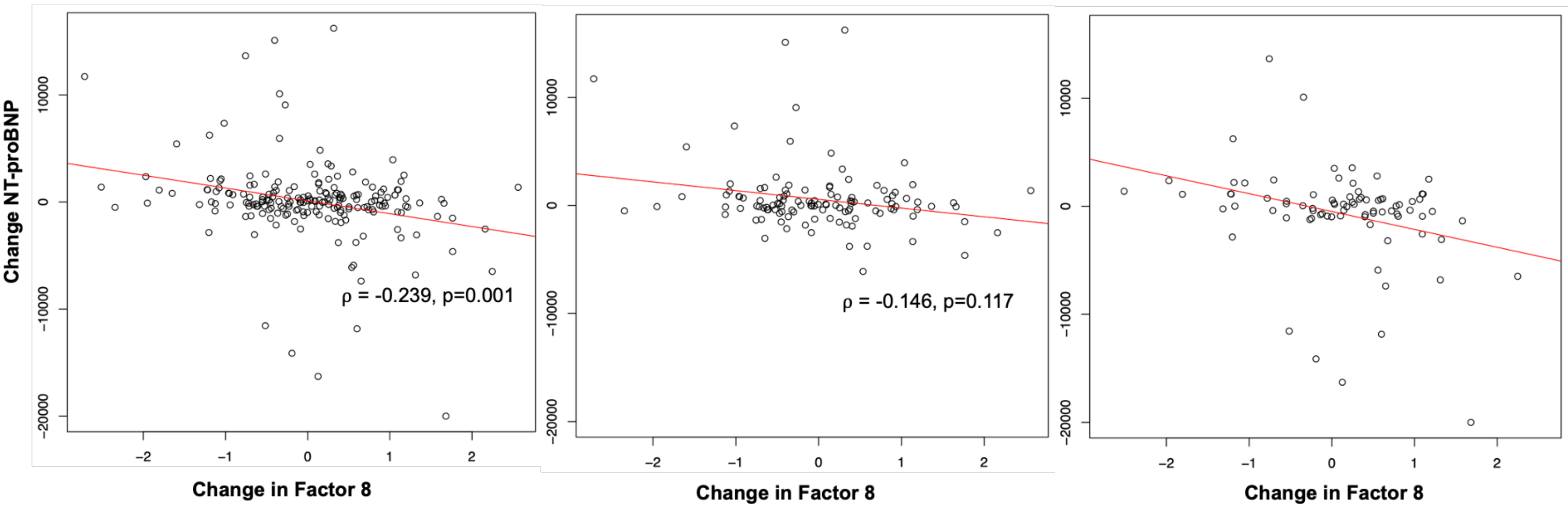
Change NT-proBNP
ρ = -0.239, p=0.001
ρ = -0.146, p=0.117
Change in Factor 8
Change in Factor 8
Change in Factor 8

f

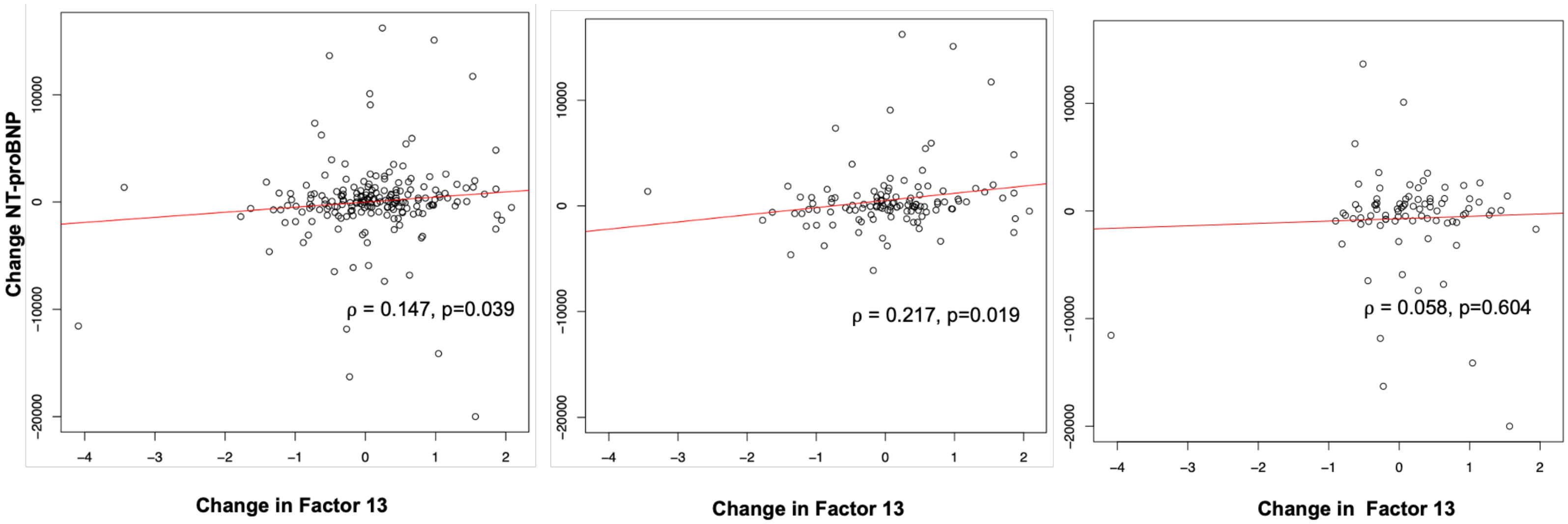
Change NT-proBNP
ρ = 0.147, p=0.039
ρ = 0.217, p=0.019
ρ = 0.058, p=0.604
Change in Factor 13
Change in Factor 13
Change in  Factor 13

**Supplementary Figure S2. Correlation plots of 90-day change in 6-minute walk distance and 90-day change in metabolite factor.** Plots are shown for the entire cohort and for individuals with and without T2DM for metabolite factors that had statistically significant associations with 90-day change in 6-minute walk distance (**a-c**).

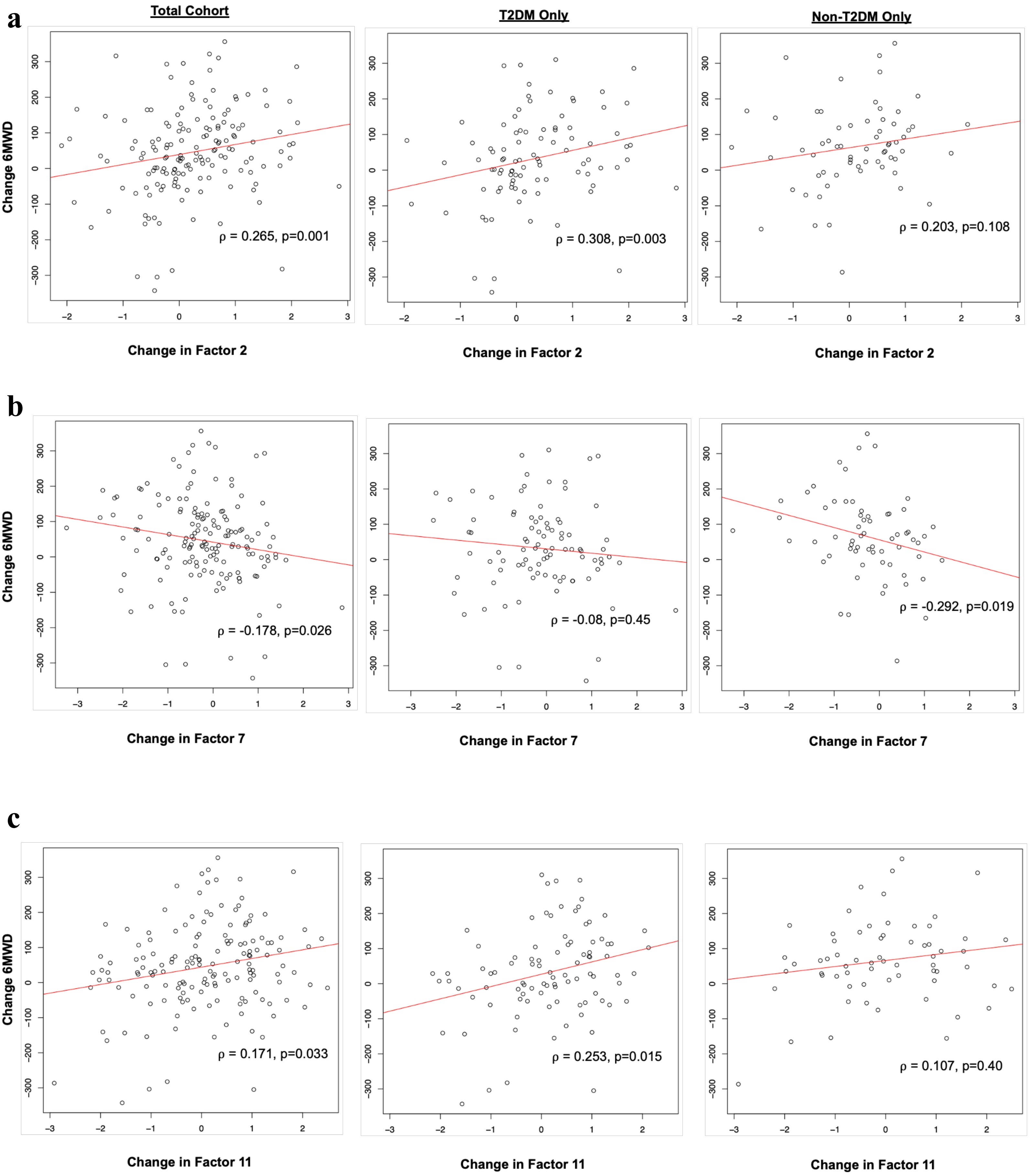

Supplement: Supplementary file 1 — Supplementary Information. [file 41598_2022_12973_MOESM1_ESM.pdf]
